# The Patient

**Published:** 1897-08

**Authors:** Chas. T. Howard

**Affiliations:** Rochester, N. Y.


THE
AMERICAN JOURNAL
—OF—
DENTAL SCIENCE.

VOL. XXXI. BALTIMORE, AUGUST, 1897. No. 4.

ARTICLE I.

### THE PATIENT.

BY CHAS. T HOWARD, D. D. S., ROCHESTER, N. Y.

Read before the Rochester Dental Society, January, 1897.

It is not with an idea of giving many new thoughts that this short paper is presented; but rather to bring to your attention in a formal way thoughts that have at times come to all of us, and some that have before now been casually touched in discussions by this society. Thoughts that flit about in a haphazard manner are often arrested and their true value found; but the chances that the true germ lying in them will be captured and its essence extracted are increased many fold by an avowed purpose to chase it down.

It is too much to expect all of our efforts to count. We must do a great deal of rattling around apparently for nothing in order to finally reach the mark. After the mark has been attained we look back and find to our amazement that what we previously considered useless beating of the bush was a necessary and vital preparation.

To successfully deal with man you must study him, his disposition, pedigrees, form, training, habits, present conditions and surroundings; just as the horseman does with the horse, only a little more so. You would be surprised how the skilled trainer of animals will "size you up," as it were, at your true natural value, as to what he can do with you, and how he can get you to do it. Of course he is not always correct, but he has rather a good basis to work on.

Thus it happens we should give attention to a few little matters outside the filling of teeth in order to reach the highest attainments in getting teeth to fill. And right here let me say you could study yourself while you are studying others. It will greatly simplify the latter.

The professional office should be a place where absolutely disinterested honesty rules. It is held that people should be honest without expecting to be paid for it. Experts are engaged in proportion to their trustworthiness. The professional man is an expert in the eyes of the people. He is paid for being disinterestedly honest. And his clientage and fees will be enhanced in proportion as the people recognize this in his opinions and advice. Every new patient weighs your advice and opinion and compares its weight with the specific gravity of disinterested honesty; determines the proportion of adulteration, such as grasping after dollars, etc., and makes a mental estimate of what it is worth. Perhaps they will decide to see you later, and perhaps not. Your treatment of the patient is absolutely in your hands, and the patient's treatment of you is largely so. You need skill in both directions, though you are not the only party who administers treatment. Part of the treatment is in your hands, the other part is on your head. The people's chief skill in securing professional services is aimed at finding this very item—disinterested honesty. And it is a sorry spectacle, indeed, that is presented when we look for the degree of skill they possess in this direction. But, nevertheless, the chances are their skill will detect your trustworthiness and their experience will find out your skill.

A person comes to you who knows little or nothing of the teeth, so far as it concerns a dentist. Be very careful how you treat such a one. First impressions count high. Whether that person ever comes to the office again or to any other dental office for nothing except extracting will depend largely on you. It may not be one that causes a large source of income to loom up in your horizon, yet remember great changes take place in a short time these days. If you treat such honestly and fairly you will find some of the least promising supports will prove your mainstay in after years. They are the ones who will measure things by very practical standards.

Be honest enough to treat all patients as though you expected to see them again, or that you expected your brother practitioner would see them. Not that you want to turn them over to another, but you had rather they would do that than to ever after hold and express an opinion derogatory of the entire profession. It is just as holden on you to sow, in all new soil presented, seed that will grow and blossom into honorable estimates and opinions of the profession at large as to produce confiding trust in your individual ability to honestly render valuable services.

A person comes to you with a tale of woe about treatment received from the hands of your fellow practitioner. Be careful what judgment you fling out or scatter in the reach of those ears. A story-monger is just as ready to retail the castings from one tongue as from another. Measure well the portrayal from those lips of the treatment given, and carefully gauge the degree of sincerity in feelings of resentment shown, and severance of former ties, before launching forth in confidential denunciation. My ultimate advice is, don't launch at all till you know the water, make a faithful effort to find and point out extenuating circumstances. We like, and are apt to trust, the man who tries to find and sees good in others. Such human jewels have little room for jealousy and the patient knows it. Too harsh words against the dentist formerly employed will often land you in as awkward position as to unite with a mother who is denouncing her child in general for some special offense. You will find the governor of the old ladies' cudgel a vascilating affair. The direction of blows is liable to change at any moment, and a hasty retreat will be in order. You can injure yourself by the way in which you show a patient their loss at the hands of another dentist. Remember this,—there is always a chance of lurking suspicions of jealousy, and a premature exposure is liable to have a wonderful back-action attachment. The patient with such things in mind is looking you through, aided by the best psychological X ray at his command. He is hunting for honesty, for skill, and for value received. He knows that he is the one to suffer if these things are wanting, so his eye is constantly on the watch. The patient is also looking for kindness, gentleness and becoming consideration. He will demand exceptional technical skill as an offset to roughness, and unimpeachable integrity to balance up bluntness. At times he is exacting; again lenient in the extreme. All his changing moods the would-be dentist must learn to meet. And like the business man, he should seek out the mysteries of artificial nature as shown forth in his fellow men.

To work a machine, oil it well, tighten securely, start it confidently; to work a man, oil abundantly, tighten cautiously, start him with fear and trembling.—*Odontographic Journal.*

